# Heat Shock Protein 70 Mediates the Protective Effect of Naringenin on High-Glucose-Induced Alterations of Endothelial Function

**DOI:** 10.1155/2022/7275765

**Published:** 2022-08-01

**Authors:** Zhihan Zhang, Hui Liu, Xiang Hu, Yikang He, Lu Li, Xinyu Yang, Changhua Wang, Mingbai Hu, Shengxiang Tao

**Affiliations:** ^1^Department of Orthopaedic Trauma and Microsurgery, Zhongnan Hospital of Wuhan University, Wuhan 430071, China; ^2^Tongji Medical College Huazhong University of Science and Technology, School of Nursing, Wuhan 430030, China; ^3^Department of Pathology and Pathophysiology, Wuhan University Taikang Medical School (School of Basic Medical Sciences), Wuhan 430071, China; ^4^Department of Breast and Thyroid Surgery, Zhongnan Hospital of Wuhan University, Wuhan 430071, China

## Abstract

Endothelial dysfunction plays a pivotal role in the development and progression of diabetic vascular complications. Naringenin (Nar) is a flavanone bioactive isolated from citrus fruits known to have *in vitro* and *in vivo* antidiabetic properties. However, whether Nar affects endothelial function remains unclear in diabetes or under high-glucose (HG) condition. Using an *in vitro* model of hyperglycemia in human umbilical vein endothelial cells (HUVECs), we found that Nar administration markedly attenuated HG-induced alterations of endothelial function, evidenced by the mitigation of oxidative stress and inflammation, the reduction of cell adhesion molecular expressions, and the improvement of insulin resistance. We also found that HG exposure significantly reduced the levels of intracellular heat shock protein 70 (iHSP70 or iHSPA1A) and the release of HSP70 from HUVECs. HSP70 depletion mimicked and clearly diminished the protective effects of Nar on HG-induced alterations of endothelial function. In addition, Nar treatment significantly enhanced iHSP70 protein levels through a transcription-dependent manner. These results demonstrated that Nar could protect HUVECs against HG-induced alterations of endothelial function through upregulating iHSP70 protein levels. These findings are also helpful in providing new therapeutic strategies that are promising in the clinical use of Nar for the treatment of diabetes and diabetic complications.

## 1. Introduction

Growing evidence has highlighted a critical role of diabetic-induced endothelial dysfunction in the development and progression of vascular complications of diabetes, including microvascular complications, such as diabetic retinopathy, nephropathy, and neuropathy, and macrovascular complications, such as coronary and peripheral artery diseases, diabetic cardiomyopathy, myocardial infarction, and so on [1–3]. It is currently believed that oxidative stress, polyol pathway, advanced glycation end products (AGE) and its receptor (RAGE), protein kinase C (PKC) signaling pathway, and some kinds of cytokines are all involved in the pathophysiological processes of endothelial dysfunction [3–5]. Although the research on the pathogenesis of endothelial dysfunction has achieved considerable advances, the novel mechanisms behind endothelial dysfunction remain to be further elucidated.

Heat shock protein 70 (HSP70 or HSPA1A) is an important member of the heat shock protein family, the most highly conserved protein family in almost all organisms. As a cytoprotective molecular chaperone, it maintains protein homeostasis through regulating protein folding and degradation [6]. HSP70 can be induced to protect cells against stressful conditions. Low expression of intracellular HSP70 protein (iHSP70) is closely associated with oxidative stress, inflammation, endoplasmic reticulum (ER) stress, cell apoptosis, fatty acid oxidation, and insulin resistance [7]. Contrarily, supplementation of alfalfa-derived HSP70 in brain significantly improves insulin sensitivity [8]. In addition, a previous study has shown that hyperglycemia reduces iHSP70 and elevates extracellular HSP70 (eHSP70) concentration, leading to diabetic vasculopathies [9]. Hence, HSP70 may become a new target for therapeutic intervention of diabetic vascular complications. However, it remains largely unclear whether or how HSP70 regulates endothelial function, especially under diabetes or hyperglycemic conditions.

Naringenin (4′,5,7-trihydroxyflavanone 7-rhamnoglucoside, Nar), a citrus-derived polyphenol belonging to flavonoids, possesses antioxidant, anti-inflammatory, antineoplastic, and immunomodulatory properties [10]. It has therapeutic and preventive potency for some chronic diseases, such as cardiovascular, neurodegenerative, pulmonary, cancer, and nephropathy. Like metformin, Nar displays *in vitro* and *in vivo* antidiabetic effects by sensitizing insulin signaling in insulin-sensitive cells and tissues, inhibiting gluconeogenesis in hepatocytes, suppressing adipocyte proliferation and adipogenesis, and protecting pancreas *β*-cells from apoptosis [11, 12]. Interestingly, a previous study hints that Nar may affect endothelial function. In streptozotocin- (STZ-) induced diabetic rats, Nar supplementation prevents some abnormal changes in vascular reactivity through preserving endothelium integrity [13]. However, there is currently a lack of direct evidence to confirm the potential impacts of Nar on endothelial function. Given that HSP70 abundance and location are tightly associated with its cytoprotective effect against oxidative stress, a stressful condition contributing to endothelial dysfunction [3–5], we speculate that antioxidant activity of Nar may be related to its regulation on HSP70, by which Nar improves diabetic- or hyperglycemia-induced impairment of endothelial function.

In the present study, we found that HG exposure reduced iHSP70 abundance in human umbilical venous endothelial cells (HUVECs). In addition, HSP70 knockdown mimicked HG-induced alterations of endothelial function. Furthermore, Nar administration markedly restored iHSP70 protein levels through transcriptional activation, resulting in the alleviation of HG-induced alterations of endothelial function. This finding provided a novel mechanism for Nar action on the protection of endothelial function.

## 2. Materials and Methods

### 2.1. Reagents

Antibodies against Akt (#9272), AS160 (#2447), HSF1 (#4356), HSP70 (#4876), phospho-Akt (Thr308, #9275), and phospho-AS160 (Ser588, #8730) were obtained from Cell Signaling Technology (Beverly, MA, USA). Anti-HSF1 (phospho-S326) antibody (ab115702) and anti-*β*-tubulin antibody (T8328) were from Abcam Inc. (Cambridge, MA, USA) and Sigma-Aldrich Corp. (St. Louis, MO, USA), respectively. Naringenin (analytical standard, 52186), 3-(4,5-dimethyl-2-thiazolyl)-2,5-diphenyl-2H-tetrazolium bromide (MTT, M2128), and dihydroethidium (DHE, D7008) were acquired from Sigma-Aldrich Corp.

### 2.2. Cell Culture and Treatment

HUVECs (#8000) were obtained from ScienCell Research Laboratories (Carlsbad, CA, USA) and cultured in Endothelial Cell Medium (#1001) as recommended by the company. To observe the impact of HG on endothelial function, HUVECs were incubated with a medium supplemented with 30 mM glucose (high glucose, HG) in the presence or absence of the desired concentration of Nar for the desired time. A medium supplemented with 5.5 mM glucose and 24.5 mM mannitol (normal glucose, NG) acted as a control. Mannitol was used to eliminate the potential impact of osmotic pressure on endothelial function. Medium was prepared as described previously [14]. Each group consisted of only one culture dish, and all experiments were repeated at least three times independently (*n* = 3). All cells were grown in an incubator at 37°C under a humidified atmosphere containing 5% CO_2_ and 95% air.

Nar with a purity ≥95% (analytical standard) was dissolved in dimethyl sulfoxide (DMSO). The same volume of DMSO was used as a negative control.

### 2.3. Determination of Glucose Uptake and PKA Activity

Glucose Uptake Assay Kit (Colorimetric, ab136955, Abcam) and PKA Kinase Activity Kit (ab139435, Abcam) were used to measure glucose uptake and PKA activity in HUVECs, respectively, according to the manufacturer's protocols.

### 2.4. Determination of Cell Damage

Cell damage was evaluated by cell viability and lactate dehydrogenase (LDH) release. MTT assay was performed to measure cell viability as described previously [14]. LDH cytotoxicity assay kit (PI88953, Thermo Scientific) was used to determine LDH levels in the culture media according to the manufacturer's instructions.

### 2.5. Determination of Oxidative Stress and Inflammation

Oxidative stress was evaluated by dihydroethidium (DHE) staining and malondialdehyde (MDA) contents in HUVECs. DHE staining for superoxide was performed as described previously [14]. The intracellular MDA levels were measured by using a lipid peroxidation (MDA) assay kit (colorimetric/fluorometric) (ab118970, Abcam) according to the manufacturer's protocol.

Intracellular nuclear transcription factor-*κ*B (NF-*κ*B) activity and interleukin-6 (IL-6) levels in the culture media were used to monitor cell inflammation in HUVECs. NF-*κ*B activity was determined by a luciferase assay as described previously [14]. IL-6 concentration in the culture media was measured using a Human IL-6 ELISA Kit (ab178013, Abcam) according to the manufacturer's protocol.

### 2.6. siRNA and Transfection

The siRNA targeting human *Hspa1a* (NM_005345.6) was synthesized by QIAGEN China (Shanghai) Co., Ltd. (Shanghai, China). The most effective sequences of siRNA and its scrambled control used in this study were as follows: *Hspa1a*, 5′- GGGCCATGACGAAAGACAACA-3′; a control nonspecific siRNA, 5′- GCATCAAGGACGCGGAACAAA-3′. Transfection was performed with 120 pM of siRNA using Lipofectamine® RNAiMAX Transfection Reagent (Invitrogen) according to the manufacturer's protocol. The knockdown efficiency was assessed by western blot.

### 2.7. Quantitative Real-Time RT-PCR (qRT-PCR)

qRT-PCR was performed to measure the mRNA levels of intercellular cell adhesion molecule-1 (ICAM-1) and vascular cell adhesion protein 1 (VCAM-1) as described previously [14]. Primers used in this study included human ICAM-1, forward 5′-CGGCCAGCTTATACACAAGA-3′, reverse 5′-GTCTGCTGGGAATTTTCTGG-3′; human VCAM-1, forward 5′-GTTGAAGGATGCGGGAGTAT-3′, reverse 5′-GGATGCAAAATAGAGCACGA-3′; and human GAPDH, forward 5′-AGGTGAAGGTCGGAGTCAAC-3′, reverse 5′-GAGGTCAATGAAGGGGTCAT-3′. GAPDH was used for normalization and for relative quantification of gene expression using the 2−ΔΔCt method.

### 2.8. Hspa1a Promoter Activity

Luciferase reporter assay was performed to evaluate *Hspa1a* promoter activity in HUVECs as described previously [15].

### 2.9. Western Blot

Cells were lysed with a lysis buffer containing 50 mM HEPES, pH 7.6, 1% Triton X-100, 150 mM NaCl, 20 mM Na_4_P_2_O_7_, 20 mM *β*-glycerol phosphate, 10 mM NaF, 1 mM Na_3_VO_4_, 1 mM PMSF, 10 mg/ml aprotinin, and 10 mg/ml leupeptin. The lysate was mixed with 2× SDS-PAGE loading buffer and then heated at 95°C for 10 min. The proteins (50 *μ*g of sample in a total volume of 20 *μ*l) were separated by an SDS-PAGE gel, transferred to a PVDF membrane, incubated with primary antibody at 4°C for overnight, and detected with horseradish peroxidase- (HRP-) conjugated secondary antibodies by using a VersaDoc Imaging System. The protein or phosphorylated protein was normalized with tubulin or nonphosphorylated protein, respectively, and quantified by densitometry using ImageJ software (ImageJ 1.4, NIH, USA).

### 2.10. Statistical Analysis

Data were presented as the mean ± standard deviation (SD). The significance of differences between groups was analyzed using analysis of variance (ANOVA), followed by a Newman–Keuls *post hoc* test (XLSTAT2020.5, Addinsoft, NY, USA). *P* values of <0.05 were considered statistically significant.

## 3. Results

### 3.1. Nar Alleviated HG-Induced Alterations of Endothelial Function

**Figure 1:**
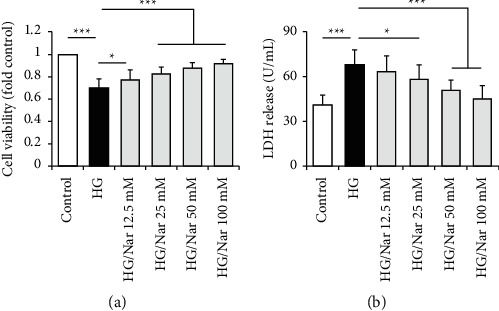
Effect of Nar on HG-induced cell damage. HUVECs were exposed to NG (5.5 mM glucose) or HG (30 mM glucose) media with or without the indicated concentration of Nar for 72 h. (a) Effect of Nar on cell viability. (b) Effect of Nar on LDH concentration in the culture media. Data are expressed as means ± SD (*n* = 4). ^*∗*^*P* < 0.05 and ^*∗∗∗*^*P* < 0.001 versus the indicated group.

Since endothelial cell injury, damage, and dysfunction affect each other [16], we firstly observed the effects of Nar on cell damage by MTT assay and measuring LDH release. HUVECs were grown in medium containing NG (5.5 mM glucose) or HG (30 mM glucose) with different concentrations of Nar (0, 12.5, 25, 50, or 100 mM) for 72 h. As shown in Figure 1, we found that Nar administration significantly restored HG-reduced cell viability (Figure 1(a)) and mitigated HG-elevated LDH concentration in the culture media (Figure 1(b)) in a dose-dependent manner, suggesting that Nar could protect HUVECs against HG-induced cell damage.

**Figure 2:**
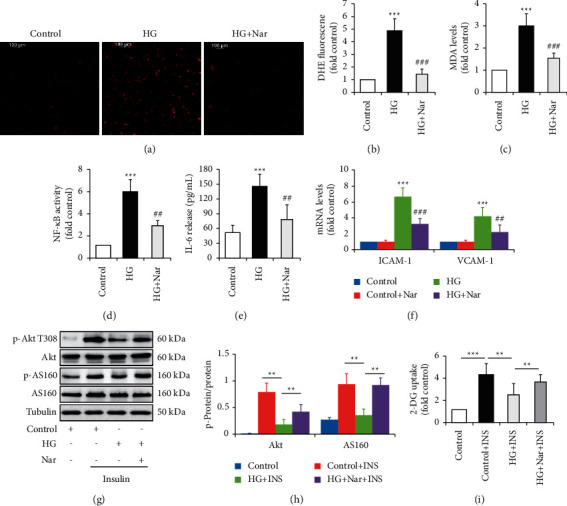
Effect of Nar on HG-induced alterations of endothelial function. HUVECs were exposed to NG (5.5 mM glucose) or HG (30 mM glucose) media with or without the 50 mM of Nar for 36 h. To observe the effect of Nar on insulin signaling, the cells were treated with or without 100 nM of insulin for 10 min. (a) Effect of Nar on ROS formation by DHE staining. (b) Quantification of relative DHE fluorescence in (a). (c) Effect of Nar on intracellular MDA concentration. (d) Effect of Nar on NF-*κ*B activity. (e) Effect of Nar on IL-6 concentration in the culture media. (f) Effect of Nar on mRNA levels of cell adhesion molecules ICAM-1 and VCAM-1. (g) Effect of Nar on insulin-stimulated phosphorylation of Akt and its downstream AS160. (h) Quantification of phosphorylated Akt and AS160 in (g). (i) Effect of Nar on insulin-stimulated 2-DG uptake. Data are expressed as means ± SD (*n* = 3). ^*∗∗*^*P* < 0.01 and ^*∗∗∗*^*P* < 0.001 versus control (NG) group or the indicated group. ^##^*P* < 0.01 and ^###^*P* < 0.001 versus high-glucose (HG) group.

Next, we figured out the potential impacts of Nar on endothelial function. HUVECs were incubated in NG or HG media in the presence or absence of 50 mM of Nar for 36 h. We found that Nar treatment markedly attenuated HG-induced oxidative stress and inflammation response, evidenced by reductions in DHE fluorescence intensity (Figures 2(a) and 2(b)), intracellular MDA concentration (Figure 2(c)), NF-*κ*B activity (Figure 2(d)), and IL-6 level in the culture media (Figure 2(e)), respectively. In addition, we also found that Nar exposure significantly suppressed HG-induced high mRNA expression of cell adhesion molecules, such as ICAM-1 and VCAM-1 (Figure 2(f)). Insulin resistance is closely associated with endothelial dysfunction and can be induced by HG-induced oxidative stress and inflammation [17]. We hence investigated the impact of Nar on insulin-stimulated signaling pathway in HG-treated HUVECs. As expected, Nar treatment greatly restored insulin-stimulated phosphorylation of Akt and its downstream AS160 (Figures 2(g)–2(h)) and increased 2-deoxy-D-glucose (2-DG) uptake (Figure 2(i)), suggesting that Nar treatment improved HG-induced insulin resistance.

Taken together, these results indicated that Nar supplementation could protect HUVECs against HG-induced alterations of endothelial function.

### 3.2. HSP70 Mediated HG-Induced Alterations of Endothelial Function

**Figure 3:**
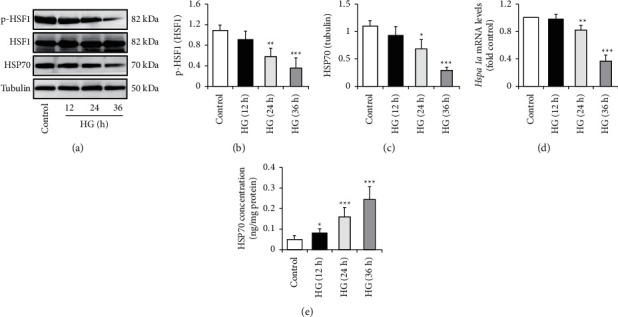
Effect of HG on iHSP70 levels. HUVECs were exposed to NG (5.5 mM glucose) or HG (30 mM glucose) media for the indicated times. (a) Effect of HG on HSF1 phosphorylation and iHSP70 levels. (b) Quantification of HSF1 phosphorylation in (a). (c) Quantification of iHSP70 levels in (a). (d) Effect of HG on *Hspa1a* mRNA levels. (e) Effect of HG on HSP70 concentration in the culture media. Data are expressed as means ± SD (*n* = 3). ^*∗*^*P* < 0.05, ^*∗∗*^*P* < 0.01, and ^*∗∗∗*^*P* < 0.001 versus control (NG) group.

Previous studies have shown the significant link between oxidative stress, inflammation, and insulin resistance, as well as type 2 diabetes and its complications [6, 7]. Moreover, these significant risk factors for diabetes and diabetic complications are related to HSP70 abundance and location [7]. We thus wanted to figure out whether HSP70 was involved in the regulation of HG-induced alterations of endothelial function. HUVECs were incubated with HG for 12, 24, or 36 h, respectively. We found that HG treatment clearly suppressed phosphorylation of heat shock transcription factor 1 (HSF1) (Figures 3(a) and 3(b)), reduced iHSP70 protein levels (Figures 3(a) and 3(c)), and increased HSP70 protein concentration in the culture media (Figure 3(e)) in a time-dependent manner, when compared with NG treatment. In addition, HG treatment also suppressed *Hspa1a* mRNA expression in a time-dependent manner (Figure 3(d)).

**Figure 4:**
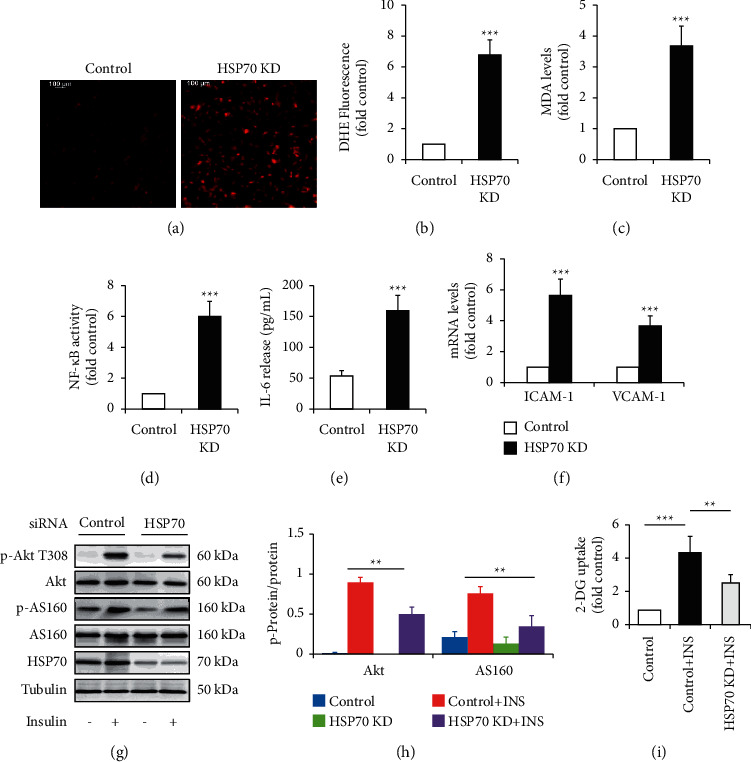
Effect of HSP70 knockdown (KD) on endothelial function. The siRNA technique was used to silence HSP70 in HUVECs. The cells were then grown in NG (5.5 mM glucose) media for 36 h. To observe the effect of Nar on insulin signaling, the cells were treated with or without 100 nM of insulin for 10 min. (a) Effect of HSP70 KD on ROS formation by DHE staining. (b) Quantification of relative DHE fluorescence in (a). (c) Effect of HSP70 KD on intracellular MDA concentration. (d) Effect of HSP70 KD on NF-*κ*B activity. (e) Effect of HSP70 KD on IL-6 concentration in the culture media. (f) Effect of HSP70 KD on mRNA levels of cell adhesion molecules ICAM-1 and VCAM-1. (g) Effect of HSP70 KD on insulin-stimulated phosphorylation of Akt and its downstream AS160. (h) Quantification of phosphorylated Akt and AS160 in (g). (i) Effect of HSP70 KD on insulin-stimulated 2-DG uptake. Data are expressed as means ± SD (*n* = 3). ^*∗∗*^*P* < 0.01 and ^*∗∗∗*^*P* < 0.001 versus siRNA control group or the indicated group.

When HSP70 in HUVECs was silenced, we found that HSP70 knockdown (KD) mimicked the impact of HG on endothelial function. Like HG treatment, HSP70 KD greatly increased oxidative stress (Figures 4(a)–4(c)) and inflammation response (Figures 4(d) and 4(e)), enhanced mRNA levels of adhesion molecules (Figure 4(f)), and inhibited insulin signaling pathway (Figures 4(g)–4(i)).

Taken together, these results suggested that HG-induced alterations of endothelial function were mediated by iHSP70.

### 3.3. Nar Enhanced HSP70 Expression in HG-Treated HUVECs

**Figure 5:**
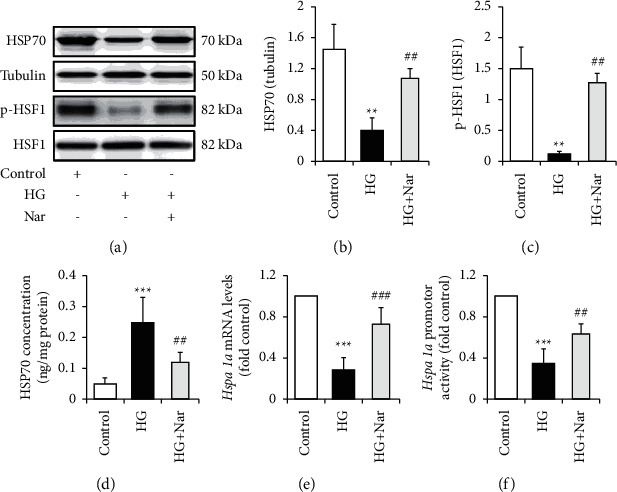
Effect of Nar on HG-reduced HSP70 levels. HUVECs were exposed to NG (5.5 mM glucose) or HG (30 mM glucose) media with or without the 50 mM of Nar for 36 h. (a). Effect of Nar on iHSP70 levels and HSF1 phosphorylation. (b) Quantification of iHSP70 levels in (a). (c) Quantification of HSF1 phosphorylation in (a). (d) Effect of Nar on HSP70 concentration in the culture media. (e) Effect Nar on *Hspa1a* mRNA levels. (f) Effect of Nar on *Hspa1a* promotor activity. Data are expressed as means ± SD (*n* = 3). ^*∗∗*^*P* < 0.01 and ^*∗∗∗*^*P* < 0.001 versus control (NG) group. ^##^*P* < 0.01 and ^###^*P* < 0.001 versus high-glucose (HG) group.

To observe the potential effect of Nar on iHSP70 levels, HUVECs were incubated with NG or HG medium in the presence and absence of 50 mM of Nar for 36 h. We found that Nar treatment clearly attenuated the deleterious effect of HG on HSP70 expression and release, evidenced by restoration of iHSP70 protein levels (Figures 5(a) and 5(b)) and HSF1 phosphorylation (Figures 5(a) and 5(c)), and suppression of HSP70 concentration in the culture media (Figure 5(d)). Importantly, HG-reduced *Hspa1a* mRNA levels (Figure 5(e)) and *Hspa1a* promotor activity (Figure 5(f)) were significantly mitigated by Nar administration. It is well known that *Hspa1a* mRNA encodes HSP70 protein and is activated at the transcriptional level by phosphorylated HSF1 [18]. These results thus suggested that Nar could improve HG-reduced HSP70 protein expression through transcriptional activation.

### 3.4. HSP70 Silence Mitigated the Protection of Nar against HG-Induced Alterations of Endothelial Function

**Figure 6:**
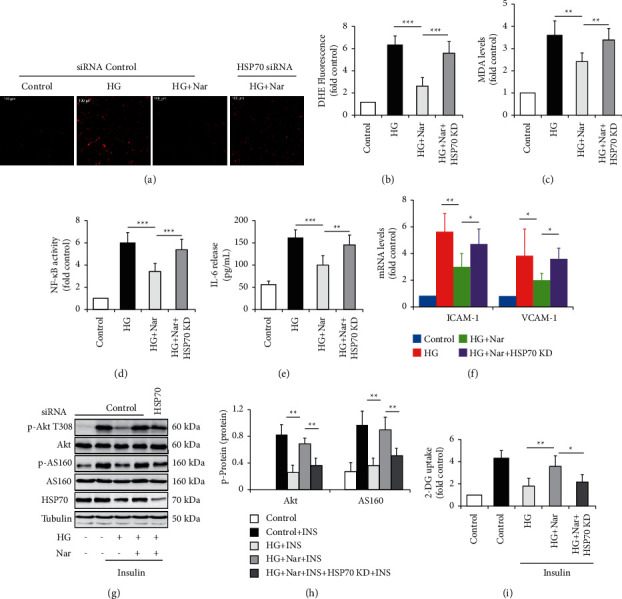
HSP70 knockdown (KD) attenuated the protective effect of Nar on HG-induced alterations of endothelial function. The siRNA technique was used to silence HSP70 in HG-treated HUVECs. The cells were then exposed to NG (5.5 mM glucose) or HG (30 mM glucose) media with or without the 50 mM of Nar for 36 h. To observe the effect of Nar on insulin signaling, the cells were treated with or without 100 nM of insulin for 10 min. (a) Effect of HSP70 KD on ROS formation by DHE staining. (b) Quantification of relative DHE fluorescence in (a). (c) Effect of HSP70 KD on intracellular MDA concentration. (d) Effect of HSP70 KD on NF-*κ*B activity. (e) Effect of HSP70 KD on IL-6 concentration in the culture media. (f) Effect of HSP70 KD on mRNA levels of cell adhesion molecules ICAM-1 and VCAM-1. (g) Effect of HSP70 KD on insulin-stimulated phosphorylation of Akt and its downstream AS160. (h) Quantification of phosphorylated Akt and AS160 in (g). (i) Effect of HSP70 KD on insulin-stimulated 2-DG uptake. Data are expressed as means ± SD (*n* = 3). ^*∗*^*P* < 0.05, ^*∗∗*^*P* < 0.01, and ^*∗∗∗*^*P* < 0.001 versus the indicated group.

To confirm whether HSP70 mediated the protective effect of Nar on HG-induced alterations of endothelial function, HSP70 was silenced in Nar-treated HUVECs. Compared with a combination of HG and Nar, we found that HSP70 KD significantly alleviated the protective effect of Nar against HG-induced oxidative stress (Figures 6(a)–6(c)), inflammation (Figures 6(d) and 6(e)), and the expressions of cell adhesion molecules (Figure 6(f)). HSP70 KD also diminished insulin sensitivity improved by Nar administration (Figures 6(g)–6(i)). These results strongly suggested that the protective effect of Nar against HG-induced alterations of endothelial function was mediated by iHSP70.

## 4. Discussion

Previous studies have evidenced that both MTT assay and LDH release are rapid, sensitive, and reliable quantitative methods to assess endothelial cell damage [19, 20]. Using these methods, we firstly demonstrated that HG significantly decreased cell viability and increased LDH release (Figure 1), which are consistent with a previous study suggesting that HG contributes to endothelial damage [20]. Interestingly, Nar protected against HG-induced endothelial damage in a dose-dependent manner (Figure 1), indicating that Nar may benefit on HG-induced impairment of endothelial function.

Endothelial dysfunction is usually defined as alterations of the endothelial phenotype characterized by increased oxidative stress, elevated expression of proinflammatory factors, increased expression of ICAM-1 and VCAM-1, insulin resistance, reduced nitric oxide (NO) bioavailability, abnormal vasoreactivity, and so on [21, 22]. In the present study, we clearly observed the protective effect of Nar on HG-induced endothelial dysfunction (Figures 1 and 2), although we did not investigate the impacts of Nar on NO bioavailability and vasoreactivity. We also confirmed that HG decreased the expressions of HSP70 protein and mRNA within HUVECs and increased HSP70 concentration in the culture media (Figure 3). Nar treatment significantly mitigated the inhibitory effect of HG on HSF1 phosphorylation, accompanied with the improvement of *Hspa1a* mRNA expression and *Hspa1a* promotor activity (Figure 5). In addition, HSP70 depletion mimicked HG-induced endothelial dysfunction and clearly mitigated the protective effect of Nar on HG-induced endothelial dysfunction (Figure 6). Given that phosphorylated HSF1 is responsible for the activation of *Hspa1a* transcription [18], our findings suggested that Nar promoted iHSP70 expression *via* a HSF1-dependent mechanism, by which Nar protected HUVECs against HG-induced endothelial dysfunction.

It is well known that HSP70 functions as a cytoprotective molecular chaperone. The alterations of HSP70 abundance and location are tightly associated with insulin resistance, diabetes, and other metabolic diseases [7]. Under stress conditions, such as hyperglycemia [9], iHSP70 is translocated to the circulation or culture media to form eHSP70 [23]. In contrast to the classical anti-inflammatory role of iHSP70, eHSP70 acts as proinflammatory factor [23]. It has been proven that both low iHSP70 abundance and high eHSP70 levels contribute to the progression of oxidative stress, inflammation, and insulin resistance in patients with obesity and diabetes [7]. However, few studies have addressed how HSP70 is regulated in endothelial cells and whether its protein level affects endothelial function. In the present study, we demonstrated that HG decreased iHSP70 expression and increased eHSP70 levels (Figure 3). HSP70 depletion generated the similar effect on endothelial function as HG did (Figure 4). These findings supported the possibility that HSP70 mediated the harmful effect of HG on endothelial function. Namely, HG-reduced iHSP70 abundance and HG-elevated eHSP70 levels cause the reduction of cytoprotective effects and the enhancement of detrimental effects respectively, leading to endothelial dysfunction.

HSF1 is a transcriptional activator of the HSP70 gene [24, 25]. Inactive HSF1 monomer is mainly located in the cytoplasm. Upon phosphorylation, HSF1 undergoes trimerization, translocates to the nucleus, and then activates *Hspa1a* transcription by binding to site-specific heat shock elements present in the *Hspa1* promoter sequence [18, 24, 25]. It is noteworthy that HSF1 can be phosphorylated by multiple signaling pathways including PKA [18, 26, 27]. In the present study, we found that HG significantly inhibited PKA activity in HUVECs (Supplementary Figure 1), which is consistent with a previous finding obtained in human retinal endothelial cells [28]. Thence, it may be possible that HG-suppressed PKA activity reduced HSF1 phosphorylation leading to a reduction of iHSP70 levels in HG-treated HUVECs. However, more studies, such as interfering PKA activity or signaling pathway, are required to further confirm the modulation of PKA on HSP70 protein levels.

Previous studies have proven the *in vitro* and *in vivo* antidiabetic properties of Nar [11, 12]. In addition, several polyphenols have also been found to protect against diabetes-related or HG-induced endothelial dysfunction in part by inhibiting oxidative stress [29]. However, it is still unclear whether Nar affects endothelial dysfunction existing in diabetes or under HG condition, although Nar displays antioxidative and anti-inflammatory properties [10]. In the present study, our results confirmed the protective effect of Nar against HG-induced endothelial dysfunction (Figures 1 and 2). Importantly, this benefit impact was markedly attenuated by HSP70 depletion (Figure 6). Combined with our results showing the positively regulation of Nar on iHSP70 protein levels (Figure 5) and HSP70 depletion-induced endothelial dysfunction (Figure 4), we could conclude that iHSP70 mediated the protective effect of Nar on HG-induced endothelial dysfunction. As for the mechanism underlying Nar regulation on HSP70 levels, we speculated that it might be related to the increase of PKA activity induced by Nar treatment (Supplementary Figure 1). Indeed, a previous study has proven that Nar could increase intracellular cAMP content and PKA activity [30]. Thence, Nar might promote iHSP70 expression through a PKA-activated HSF1 phosphorylation [18, 31]. Furthermore, the inhibitory effects of Nar on eHSP70 release (Figure 5(d)) also helpfully reduced the harmful effect of HG on endothelial function. However, the underlying mechanism by which Nar inhibits eHSP70 formation is still required to be carefully evaluated.

Of note, the relationships between oxidative stress, inflammation, and HSP70 abundance and location are very complicated. It has been documented that enhanced oxidation and inflammation are closely interlinked processes in diabetes and diabetic complications, or under HG condition, which can be effectively controlled by HSP70 [7, 32]. On the other hand, reduced iHSP70 directly or indirectly activates NF-*κ*B-dependent inflammatory pathways and contributes to the formation of reactive oxygen species (ROS). Besides, the inflammatory response stimulates eHSP70 formation and suppresses iHSP70 expression [32]. Thus, it should be a promising stratagem to retard the development and progression of endothelial cell-related complications in diabetes, through restoring iHSP70 abundance to break the vicious circle between oxidative stress, inflammation, and iHSP70 reduction. Likely, Nar prevents this vicious circle by regulating iHSP70 levels and eHSP70 formation, by which Nar improves HG-induced impairment of endothelial function.

In the present study, we found that Nar markedly improved HG-induced insulin resistance, demonstrated by the improvements of insulin-stimulated 2-DG uptake and phosphorylation of Akt T308 and its downstream AS160 (Figures 2(g)–2(i)). Additionally, HSP70 depletion not only induced insulin resistance (Figures 2(g)–2(i)) but also significantly attenuated the protective effects of Nar on HG-reduced insulin sensitivity (Figures 4(g)–4(i)). These findings suggest for the first time that HSP70 plays a critical role in mediating Nar action on insulin signaling.

Endothelial dysfunction is closely associated with insulin-resistant states, including diabetes and diabetic complications [21]. Insulin resistance is characterized by defects of insulin signaling and reduction of glucose uptake in peripheral insulin-sensitive cells or tissues. Glucose uptake, a highly regulated process mediated by cell surface localization of glucose transporters (GLUTs) [33], is important for the survival and growth of endothelial cells, due to its reliance on glycolysis as an energy source [34]. Interestingly, HUVECs lack the expressions of GLUT2, GLUT4, and GLUT5 mRNA [35, 36]. Previous study has shown that glucose uptake in HUVECs is mainly mediated by GLUT1 [36]. Defects of insulin signaling such as PI3K inhibition and AKT knockdown suppressed insulin-induced GLUT1 translocation [37, 38], leading to a reduction of glucose uptake. In the present study, we only demonstrated the effects of Nar and iHSP70 on insulin-dependent glucose uptake in HUVECs (Figures 2(i), 4(i), and 6(i)). Thence, more studies are required to identify GLUTs isoforms involved in this process.

## 5. Conclusion

Although the underlying signaling pathway is needed to be further elucidated, we found that Nar could upregulate HSP70 protein levels through a transcriptional mechanism, leading to the improvement of HG-induced endothelial dysfunction. Of course, this HSP70-mediated protective effect should be investigated in further *in vivo* studies. The potential effect of Nar on diabetic vascular complications also deserves further observation. A full understanding of Nar action is helpful in providing new strategies for its clinical treatment for diabetes and diabetic complications.

## Data Availability

The data that support the findings of this study are available upon request from the corresponding author, Shengxiang Tao (zn-taoshengxiang@163.com).

## Supplementary Materials

Supplementary Materials
*Supplemental Figure 1*. Nar restored HG-reduced PKA activity. HUVECs were exposed to NG (5.5 mM glucose) or HG (30 mM glucose) media with or without the 50 mM of Nar for 36 h. PKA activity was determined by PKA Kinase Activity Kit from Abcam. Data are expressed as means ± SD (*n* = 3). ^*∗∗*^*P* < 0.01 and ^*∗∗∗*^*P* < 0.001 versus the indicated group.
