# Supplementary material for: Heat Shock Protein 70 Mediates the Protective Effect of Naringenin on High-Glucose-Induced Alterations of Endothelial Function
Source: Int J Endocrinol. 2022 Aug 1;2022:7275765. doi: 10.1155/2022/7275765 (PMC9359828; doi:10.1155/2022/7275765)
Supplement: Supplementary Materials — Supplemental Figure 1. Nar restored HG-reduced PKA activity. HUVECs were exposed to NG (5.5 mM glucose) or HG (30 mM glucose) media with or without the 50 mM of Nar for 36 h. PKA activity was determined by PKA Kinase Activity Kit from Abcam. Data are expressed as means ± SD (n = 3). ∗∗P < 0.01 and ∗∗∗P < 0.001 versus the indicated group. [file 7275765.f1.docx]

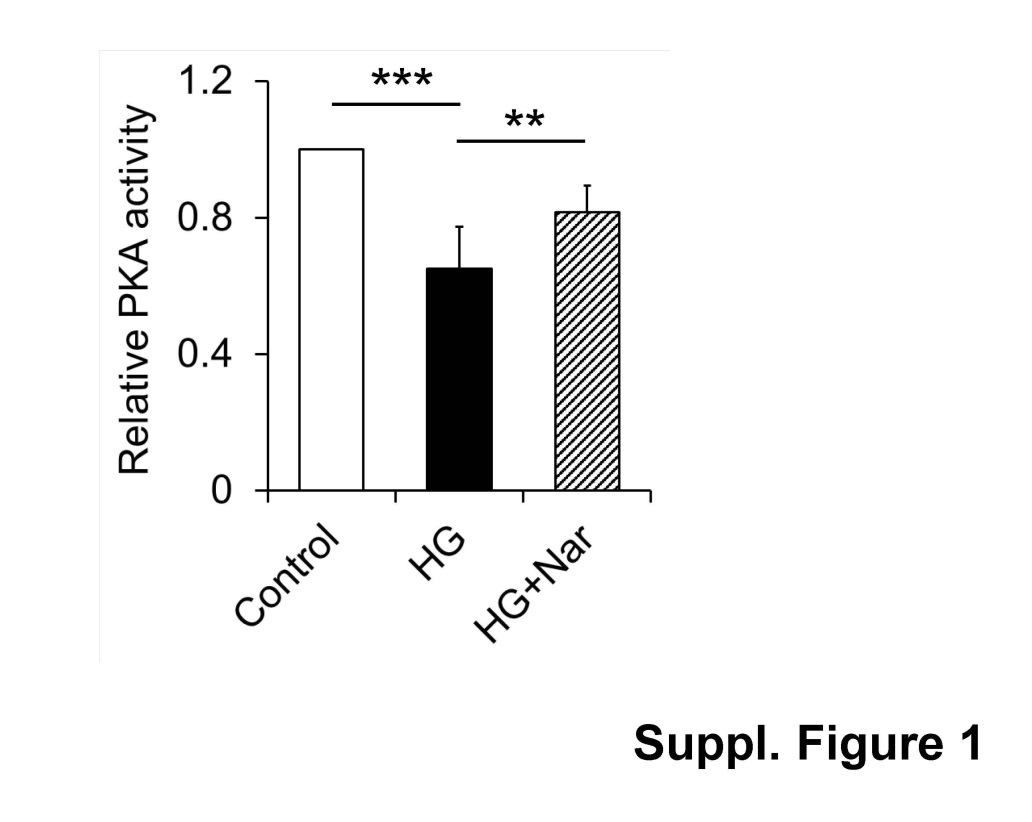


**Supplemental Figure 1**: Nar restored HG-reduced PKA activity. HUVECs were exposed to NG (5.5 mM glucose) or HG (30 mM glucose) media with or without the 50 mM of Nar for 36 h. PKA activity was determined by PKA Kinase Activity Kit from Abcam. Data are means ± SD (n = 3). **P < 0.01, ***P < 0.001 vs. the indicated group.
